# Exploring the role of underrepresented populations in polygenic risk scores for neurodegenerative disease risk prediction

**DOI:** 10.3389/fnins.2024.1380860

**Published:** 2024-05-27

**Authors:** Kathryn Step, Carene Anne Alene Ndong Sima, Ignacio Mata, Soraya Bardien

**Affiliations:** ^1^Division of Molecular Biology and Human Genetics, Faculty of Medicine and Health Sciences, Stellenbosch University, Cape Town, South Africa; ^2^Genomic Medicine, Lerner Research Institute, Cleveland Clinic Foundation, Cleveland, OH, United States; ^3^South African Medical Research Council/Stellenbosch University Genomics of Brain Disorders Research Unit, Stellenbosch University, Cape Town, South Africa

**Keywords:** polygenic risk score (PRS), genome-wide association study (GWAS), neurodegenerative disease, underrepresented populations, susceptibility variants

## Introduction

Neurodegenerative diseases (NDs) are characterized by the progressive loss of neuronal function and structure (Lamptey et al., 2022). Additionally, neurodegeneration is associated with neural networks, synaptic dysfunction, and the accumulation of physiochemically altered proteins in the brain (Hoover et al., 2010; Milnerwood and Raymond, 2010). NDs affect ~15% of the global population making them the leading cause of cognitive and physical disability worldwide (Feigin et al., 2020). Moreover, the prevalence of several of these diseases increases with age, therefore the global trend toward an increased life expectancy may also result in an increased burden of age-related NDs such as Alzheimer's disease (AD) and Parkinson's disease (PD; GBD 2016 Parkinson's Disease Collaborators, 2018). Of all the NDs, AD and related dementias and PD have been the most studied and genetically characterized. For this reason, these two disorders are highlighted in this article.

On an international scale, the majority of genetic research still primarily focuses on populations of European ancestry resulting in the relative absence of “non-European” populations (also known as underrepresented populations; URPs) from large-scale genomic study cohorts and subsequently, GWAS (Knerr et al., 2011). Many countries in the “Global South” are considered URPs, including but not limited to countries in Africa, Asia, Caribbean, Latin America, and Oceania (excluding Australia and New Zealand; Fatumo et al., 2022; Schumacher-Schuh et al., 2022; Bhattacharya et al., 2023). For the purposes of this article, URPs include countries in Asia such as Japan and Korea, since they have also been comparatively underrepresented in genetic studies.

There is an increased prevalence of PD and AD in URPs (GBD 2021 Nervous System Disorders Collaborators, 2024). Moreover, AD and related dementias are in the top five most prevalent nervous system disorders across URPs (GBD 2021 Nervous System Disorders Collaborators, 2024). It is predicted that worldwide there will be 65.7 million people in 2030 and 115.4 million people in 2050 living with dementia, with 63 and 71% living in URPs in 2030 and 2050, respectively (Ferri et al., 2005; Prince et al., 2013).

AD and PD have a complex etiology and while age is a common risk factor of certain disease development, many are considered multifactorial diseases with both environmental and genetic components. Exposure to environmental pollutants such as neurotoxic metals (e.g., lead, manganese, and mercury) and pesticides have been associated with disease pathogenesis by increasing beta-amyloid peptides, the phosphorylation of Tau protein, and the aggregation of α-synuclein (Chin-Chan et al., 2015). Although there have been great advances in elucidating the genetic architectures of AD and PD, the complete genetic etiology has yet to be defined further highlighting the complexity of these diseases. However, studies have shown that these diseases have a significant genetic component through the identification of rare variants with large effect in causal genes in familial cases, as well as common variants with smaller effect through association analysis studies in sporadic cases (Perrone et al., 2021).

Genetic risk variants, commonly known as susceptibility variants, that are statistically associated with the risk of developing a particular disease or trait, are identified using genome-wide association studies (GWAS; Uffelmann et al., 2021). In contrast to linkage analysis studies that utilize multiplex family datasets, GWAS use unrelated case-control datasets to perform an association analysis (Tan et al., 2014). These studies have revealed the polygenic architecture of several common complex disorders including NDs. For example, more than 90 and 75 genetic loci have been linked to PD (Nalls et al., 2019) and AD development (Bellenguez et al., 2022), respectively.

## GWAS on NDs in URPs

**Figure 1:**
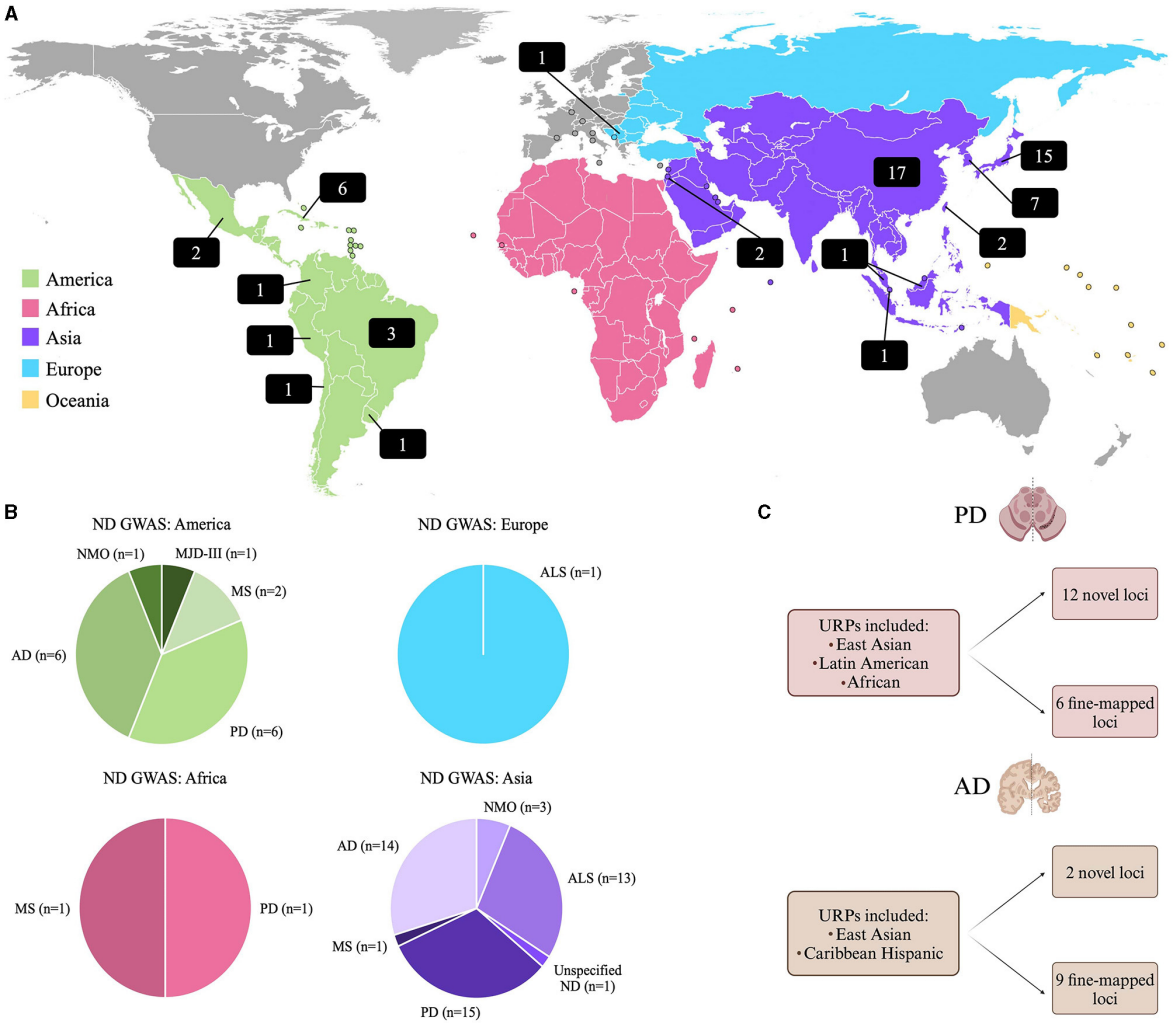
**(A)** World map showing the distribution of GWAS on NDs in URPs. The numbers in the black blocks indicate the number of GWAS publications that included participants from URPs in either a discovery or replication cohort. **(B)** A summary of the GWAS Catalog results for NDs indicating from which continent the URPs included in the GWAS originated from [(Sollis et al., 2023); all data as of September 11th, 2023]. **(C)** A summary of two recent multi-ancestry meta-analysis and fine-mapping studies on PD (Vaswani et al., 2020) and AD (Hamid et al., 2021) that identified novel loci and fine-mapping of causal loci by leveraging diverse haplotype blocks in URPs (Kim et al., 2022; Lake et al., 2023). AD, Alzheimer's disease; ALS, Amyotrophic Lateral Sclerosis; MJD-III, Machado-Joseph Disease; MS, Multiple Sclerosis; ND, Neurodegenerative disease unspecified; NMO, Neuromyelitis optica; PD, Parkinson's disease.

Historically, GWAS have almost solely focused on participants of European ancestry (Lewis and Vassos, 2020), where ~94.8% of previously conducted GWAS included European participants, while only 5.2% included all other population groups combined (Mills and Rahal, 2020). Since current risk prediction methods require an individual's genetic ancestry to be similar to the ancestries from a well-powered GWAS study, from which effect sizes can be estimated (Martin et al., 2019), this significantly limits the applicability of PRS done on Europeans, to URPs. A review of the GWAS Catalog (Sollis et al., 2023), revealed that as of September 11th, 2023, there were 355 ND GWAS studies reported with only 18% (*n* = 63) of these studies including individuals from URPs. This data is summarized in Figure 1, and a complete list of these studies is provided in Supplementary Table 1.

Moreover, there is a lack of diversity in ND research overall, including clinical trials, epidemiology studies, neuroimaging studies, and case studies (Vaswani et al., 2020; Franzen et al., 2022). This may be due to limited neurology services in URP regions, such as Africa, leading to people with NDs being undiagnosed or mis-diagnosed (Hamid et al., 2021). The disproportionate exclusion of ethnically diverse individuals in ND research and clinical trials can be attributed to the challenges with the diagnosis process, recruitment strategies, study enrolment, and participant retention practices (Gilmore-Bykovskyi et al., 2019).

## Polygenic risk scores

Originally, risk prediction for complex diseases relied heavily on information on the family's medical history. More recently, polygenic risk scores (PRS) can be calculated and used to estimate an individual's lifetime risk of developing a particular disease. This is based on the combination of genetic susceptibility variants that they carry contributing either to an increased or decreased disease risk (Lewis and Vassos, 2020). The genetic susceptibility variants and the effect size estimates utilized in a PRS analysis are typically identified through GWAS. However, it should be noted that PRS only explains a small percentage of the risk, which needs to be complemented with a combination of factors including family history, environmental exposures, lifestyle factors, and clinical features, such as loss of smell and sleep disturbances as prodromal symptoms of disease (So et al., 2011; Seifan et al., 2019). It has been reported that PRS based on common genetic variants accounts for ~7% of the variance in AD risk in European populations (Leonenko et al., 2021) and 2.2% variance for PD risk in the Latino population (Loesch et al., 2022). Ultimately, increasing representation from URPs may improve the predictive validity by capturing a broader range of genetic variation associated with the disease.

As an emerging precision medicine tool, accurate disease risk prediction could inform clinicians at different points of disease trajectory from providing information regarding the most efficient prevention strategies, to targeted screening interventions and disease diagnosis, as well as provide improved clinical management (Lewis and Vassos, 2020; Corpas et al., 2022). While there is evidence for the clinical utility and validity of PRS, there are still concerns relating to the transferability of these results across populations, the accuracy of disease prediction in heterogenous diseases, and whether PRS disclosure does indeed affect morbidity and mortality rates (Kumuthini et al., 2022). An individual's genetic ancestry has a significant impact on risk prediction. The distinct genetic architectures of different populations influence PRS performance due to differences in population-specific linkage disequilibrium patterns and allele frequencies (Sirugo et al., 2019). Additionally, the transferability of PRS across different populations can be questioned, as prevalence rates and genetic risk variants vary between populations (Kachuri et al., 2024). Therefore, a PRS developed using one ancestry may not be applicable to individuals of another ancestral group (Fatumo et al., 2023).

To address the issue of transferability, it is essential to develop methods and models that are inclusive of diverse populations and incorporate ancestry-specific genetic information (Cavazos and Witte, 2021; Fatumo et al., 2023). The incorporation of local ancestry in GWAS, using software such as TRACTOR (Atkinson et al., 2021), has shown promising results in identifying ancestry-specific risk loci which in turn can be used for PRS calculations (Swart et al., 2021). Recent efforts, such as PRS-CSx (Ruan et al., 2022) and BridgePRS (Hoggart et al., 2023), aim to improve PRS transferability by leveraging multi-ethnic datasets and accounting for population stratification and admixture. However, accurately predicting disease risk in admixed populations remains a challenge due to their complex genetic composition (Duan et al., 2018; Swart et al., 2021). Therefore, the general PRS calculation method that utilizes only a single training (i.e., discovery) population would not be adequate to predict polygenic risk in admixed individuals since the choice of the discovery cohort to use is unknown. To tackle this issue, approaches like using a linear combination of PRS based on multiple training datasets show promise in improving prediction accuracy in admixed populations (Márquez-Luna and Loh, 2017). However, there are still limitations with the current methods, and it remains vital that the development of novel methods should go hand-in-hand with increasing the diversity of study cohorts.

## Steady increase in genomic data from URPs

There has been a steady increase in the number of GWAS including URPs, specifically individuals of Asian ancestry (Mills and Rahal, 2020). This has been achieved through the implementation of large prospective population-based biobanks such as the China Kadoorie Biobank (Chen et al., 2011), BioBank Japan (Nagai et al., 2017) and major genome projects such as the Singapore 10K Genome project (Precise Health Research Singapore, 2023). In the future, the application of PRS in African countries may be feasible through initiatives like the Three Million African Genome (3MAG) project (Wonkam, 2021).

Furthermore, as the field progresses, there is an increase in the establishment of disease-specific consortia. For example, the Multi-Partner Consortium to Expand Dementia Research in Latin America (ReDLat) is an initiative aiming to increase dementia research in Latin America and the Caribbean by combining clinical and genomic data (Ibanez et al., 2021). Likewise, the European Alzheimer & Dementia Biobank (EADB) consortium brings together various European GWAS consortia focused on investigating the genetic architecture of AD through a GWAS meta-analysis study (Bellenguez et al., 2022). Notably, collaborative efforts in the field of PD genetics have been particularly promising. Consortia such as the Latin American Research Consortium on the Genetics of Parkinson's Disease (LARGE-PD; Zabetian and Mata, 2017), the Genetic Epidemiology of Parkinson's Disease consortium (GEoPD; Farrer et al., 2021), the International Parkinson's Disease Genomics Consortium (IPDGC) Africa (Rizig et al., 2021), and the East Asian Parkinson's Disease Genomics Consortium (EAPDGC; Mok, 2021) were formed to investigate the genetic etiology of PD. Many of these consortia are in the process of integrating into a global effort to support the Global Parkinson's Genetics Program (GP2).

GP2 is an international collaboration investigating the genetic etiology of PD and making the knowledge globally relevant by increasing representation of URPs in PD research (Towns et al., 2023). GP2's goal is to collect standardized clinical data and genotype more than 200,000 participants with a minimum of 50,000 individuals from URPs for use in association studies. A recent GP2 GWAS, involving African and African admixed participants (not yet included in GWAS Catalog), revealed a novel ancestry-specific genetic risk factor for PD in *GBA1* (rs3115534; Rizig et al., 2023). This finding highlights the *value of leveraging genetic diversity* to detect novel disease associations. Collaborative large-scale international efforts, such as GP2, show promise in correcting the significant imbalance of European population biases in GWAS, thereby ultimately improving the accuracy of disease prediction in URPs.

Additional key factors to aid in the analysis of admixed populations and allow for the inclusion of URPs in GWAS include suitable genotyping arrays, reference panels for phasing and imputation, ancestry inference, and robust GWAS QC metrics tailored for URPs (Atkinson et al., 2021; Yang et al., 2023). Innovations in methodological development are ongoing to address the complexities of genetic studies involving URPs. Anticipated advancements in statistical methods, software tools, and genetic reference panels are poised to play pivotal roles as research endeavors increasingly embrace representative sample recruitment and the global population's growing diversity (Sariya et al., 2019; Tan and Atkinson, 2023).

## Value of increasing URPs in genomic studies

It has been widely acknowledged that increasing diversity in genetic research will aid in understanding disease etiology, ultimately improving the effectiveness of genomic medicine (Peterson et al., 2019). However, results from genetic research do not translate equally across populations, further highlighting the importance of including URPs in genetic research. For example, the apolipoprotein E (*APOE*) gene is a well-studied risk factor for AD and related dementias, however, its prevalence and influence on disease risk varies significantly among ancestral groups (Farrer et al., 1997; Tang et al., 2001). In East-Asian populations, *APOE* ε4 is the strongest risk effect, however, in African populations, it is the lowest risk effect, trending toward a protective effect (Hendrie et al., 2014; Rajabli et al., 2022). Moreover, many URPs are comprised of multiway admixed individuals with genomic architecture that consists of smaller haplotype blocks than those of the European genomes (Fatumo et al., 2023). Haplotype structure is useful in inferring information relating to gene flow and population structure (Shipilina et al., 2023).

Consequently, the inclusion of URPs can aid in fine-mapping known disease loci to improve post-GWAS functional studies by reducing the number of candidate variants prioritized. Additionally, the alleles observed in diverse admixed populations can facilitate the identification of novel population-specific disease risk variants (Rizig et al., 2023) and reveal disease risk variants for various populations, simultaneously (Swart et al., 2022). This utility was highlighted in recent multi-ancestry GWAS studies on PD and AD (Figure 1C). A PD meta-analysis identified 12 potentially novel loci and six putative causal variants (Kim et al., 2022). For AD, a similar approach was employed to leverage diverse haplotype structures to aid in variant identification and causal loci fine-mapping, where two novel disease loci were identified and nine loci were fine-mapped (Lake et al., 2023).

In addition to the unique ancestral makeup, there are a range of varying socio-economic factors affecting URPs, resulting in observed differences in disease prevalence and phenotypes. For example, in comparison to European populations, Latin American populations are 1.5 times more likely to develop AD and related dementias (Prince et al., 2013; Matthews et al., 2018). Factors affecting the increased prevalence may include lifestyle, work exposures, economic access to resources, access to healthcare, education on preventive and precautionary actions as well as living conditions (Epping-Jordan et al., 2005; Babulal et al., 2019). Consequently, the inclusion of URPs in genomic studies is vital to investigating the underlying genetic etiology of these diseases through the identification of pathogenic and susceptibility variants, in addition to identifying the social and environmental factors resulting in an increased or decreased disease risk.

It has been shown that there is limited clinical utility for PRS in non-European populations when calculating risk prediction using a European cohort as the discovery dataset (Saffie-Awad et al., 2023). This further highlights the importance of large-scale diverse GWAS and potentially improving PRS model prediction at a variant level by incorporating local ancestry proportions, and at a population level by incorporating global ancestry proportions (Márquez-Luna and Loh, 2017). Globally, to an extent, most individuals are multiway admixed, therefore the development and implementation of more inclusive GWAS and PRS models will allow a more accurate risk prediction by leveraging variant effect size distributions across populations (Song et al., 2020).

## Need for integrated datasets for PRS

PRS models calculate an individual's risk of disease development using the cumulative estimated effect of all nominally associated genetic variants and not only genome-wide significant variants (Bellou et al., 2020). Ultimately, PRS uses the weighted sum of a large number of SNPs with small effect sizes. Risk allele patterns were previously examined using individual-level data where differences in allele frequency, directionality, and the magnitude of effect were observed across several ancestral populations (Saffie-Awad et al., 2023). This further highlights the allele frequency differences observed between populations as well as population-specific disease risk, emphasizing the need for diverse GWAS summary statistics to be employed in PRS models to improve risk prediction accuracy.

Due to the complexity of these diseases, there is a level of environmental risk factors that contribute independently to disease risk (Lewis and Vassos, 2020). Therefore, *integrated prediction models* using PRS with additional environmental exposures and lifestyle information enables researchers to more accurately quantify risk (Martin et al., 2019). However, cohort size, lack of population diversity in PRS research, and inconsistent clinical characteristics severely limit PRS estimates (Saffie-Awad et al., 2023). Hence, incorporation of environmental information (e.g., smoking status and pesticide exposure) as well as clinical information (e.g., age of onset and family history) can improve the risk prediction model's stability, accuracy, and sensitivity (Truong et al., 2023). This information needs to be consistently recorded and standardized between study cohorts and would need to be collected during study participant recruitment.

## Standardization of the data

The medical and biological fields have advanced greatly over the past two decades resulting in a data-rich area of research (Holmes et al., 2010). Many large publicly available repositories are used in research by compiling several sources of data including demographic data, clinical data, environmental and lifestyle information, molecular genetic data, and genealogical information (Lowrance, 2001). However, many of these repositories often have extensive clinical information without genetic data or alternatively have sufficient molecular genetic data with a limited clinical characterization of phenotypes. While there are disease-specific databases like the Accelerating Medicines Partnership- Alzheimer's Disease (AMP-AD) and the Parkinson's Disease Biomarkers Program (PDBP; Hodes and Buckholtz, 2016; Ofori et al., 2016), the majority of individuals included in these databases are from European populations, further highlighting the lack of URP representation. Ultimately, there is an increased need for *standardized databases that are representative of the global population* with sufficient quality information for each data category that can be used in PRS models for increased sensitivity and specificity to ultimately improve risk prediction accuracy.

## Recommendations

In summary, a list of the pertinent recommendations for future PRS studies is provided below:

- Inclusion of ethnically diverse individuals in research studies.
- Need for continuous advancements in statistical methods, software tools, and genetic reference panels to appropriately include URPs samples in PRS research.
- Leverage genetic data from biobanks and disease-specific consortia.
- Leverage genetic diversity to detect novel disease associations and fine-mapping of known disease loci.
- Use of integrated prediction models using PRS combined with environmental exposures, lifestyle factors, and traditional risk factors e.g., smoking status.
- Standardization of genomic databases that are representative of the global population.

## Concluding remarks

The value of including URPs in genetic research is highlighted by building capacity for genomic research on a global scale and ensuring the equitable implementation of precision medicine tools. While it has been perceived that incorporating genetically diverse populations (mainly URPs) in genetic research is challenging, there are expanding analytical approaches and algorithms to accurately account for population substructure in GWAS (Peterson et al., 2019) aiding in the detection of true genetic associations that can be utilized in risk prediction. Ultimately, the inclusion of URPs has the potential to fast-track the field of risk prediction, to aid in novel susceptibility variant detection, disease prediction, and the development of precision medicine strategies that are both applicable and accessible to all populations.

## Author contributions

KS: Conceptualization, Writing – original draft, Writing – review & editing. CN: Writing – original draft, Writing – review & editing. IM: Conceptualization, Writing – review & editing. SB: Conceptualization, Writing – review & editing.
